# Experimental Investigation for Fault Diagnosis Based on a Hybrid Approach Using Wavelet Packet and Support Vector Classification

**DOI:** 10.1155/2014/145807

**Published:** 2014-02-12

**Authors:** Pengfei Li, Yongying Jiang, Jiawei Xiang

**Affiliations:** ^1^College of Mechanical and Electrical Engineering, Wenzhou University, Wenzhou 325035, China; ^2^School of Mechanical and Electrical Engineering, Guilin University of Electronic Technology, Guilin 541004, China

## Abstract

To deal with the difficulty to obtain a large number of fault samples under the practical condition for mechanical fault diagnosis, a hybrid method that combined wavelet packet decomposition and support vector classification (SVC) is proposed. The wavelet packet is employed to decompose the vibration signal to obtain the energy ratio in each frequency band. Taking energy ratios as feature vectors, the pattern recognition results are obtained by the SVC. The rolling bearing and gear fault diagnostic results of the typical experimental platform show that the present approach is robust to noise and has higher classification accuracy and, thus, provides a better way to diagnose mechanical faults under the condition of small fault samples.

## 1. Introduction

The bearing and gear are the most critical and frequently encountered components in vast majority of rotating machinery. Their operating state directly affects the machine performance, efficiency, and life. Therefore, fault identification of rolling element bearing and gear has been the subject of extensive research.

Vibration analysis has been established as the most common and reliable method of analysis. Generally, the vibration signals can be used to detect the incipient fault of the machine components and reduce the possibility of catastrophic damage and the down time, through the on-line monitoring and diagnosis system [1, 2]. The extracted features include time domain features such as root mean square, variance, skewness, and kurtosis [3–5], frequency domain features such as content at the feature frequency and the amplitudes of frequency spectrum [6, 7], and time frequency domain features such as the statistical characteristics of short-time Fourier transform (STFT), Wigner-Viller distribution (WVD), wavelet transform (WT), and so forth [8–10]. The WT method possesses perfect local property in both time space and frequency space, and it is used widely in the region of machinery fault detection and identification [11–13]. However, the WT cannot split the high frequency band where the modulation information of machine fault is often involved in. The wavelet package transform (WPT) can overcome the difficulty. Nikolaou and Antoniadis proposed a method for the analysis of vibration signals resulting from bearing with localized defects using the wavelet packet transform [14]. Fan and Zuo combined Hilbert transform and wavelet packet transforms to extract modulating signal and detect the early gear fault [15]. Wang and Lin investigated fault signals denoising processing using wavelet packet decomposition coefficients to identify the weak fault characteristic frequency of rolling bearings under strong background noise [16]. However, these investigations did not combine intelligent fault diagnosis techniques to further recognize faults. The carefully selected vibration signals are necessary to match the theoretical fault frequency. For this reason, the advantages of WPT are not reflected.

Many intelligent classification algorithms, such as artificial neural networks (ANNs) and support vector classification (SVC), have been proposed to detect mechanical faults and recognize machine conditions [1–3]. The main difference between ANNs and SVC is in their risk minimization. In the case of SVC, structural risk minimization principle is used to minimize an upper bound based on an expected risk. In ANNs, traditional empirical risk minimization is employed to minimize the error in training of data. The difference in risk minimization leads to a better generalization performance for SVC than ANNs. Thukaram et al. [17] compared the differences between the ANNs and SVC in identifying the fault. Crampton and Mason [18] found that when the data contains noise, the fault detection using support vector machine (SVM) is more effective than other intelligent techniques. However, only ANNs or SVC can not obtain satisfactory classification results from high level ambient noise. Therefore, in recent years, more and more researchers focus on the hybrid approach using WPT and SVC for fault classification. Bin et al. [19] combined WPT and empirical mode decomposition to extract fault feature frequency and further employed ANNs to detect faults in rotating machinery. Hu et al. [20] presented a hybrid approach for bearing fault diagnosis using WPT and SVC. Xian and Zeng developed Hu's scheme for bearing fault diagnosis using WPT and hybrid SVC [21]. Shen et al. proposed a new scheme using the extraction of statistical parameters from WPT of original signals, a distance evaluation technique, and a support vector regression (SVR) based generic multiclass solver [22]. However, due to the limitation of machinery fault simulator, the fault samples used in the above investigations came from a single data source, mostly from the Case Western Reserve University official website. Therefore, the superiority of the hybrid approaches is not confirmed.

For the above reasons, this paper presents a hybrid approach for bearing and gear fault diagnosis based on a hybrid approach using wavelet packet decomposition and SVC. To validate the proposed method, we carry out experimental investigations using the machinery fault simulator (MFS-MG). A large number of experimental data is collected for bearing and gear under different working conditions. Our test results have shown that the proposed approach is effective and can further detect mechanical faults with an agreeable precision.

## 2. Wavelet Packet Decomposition and Support Vector Classification Diagnostic Principles

**Figure 1:**
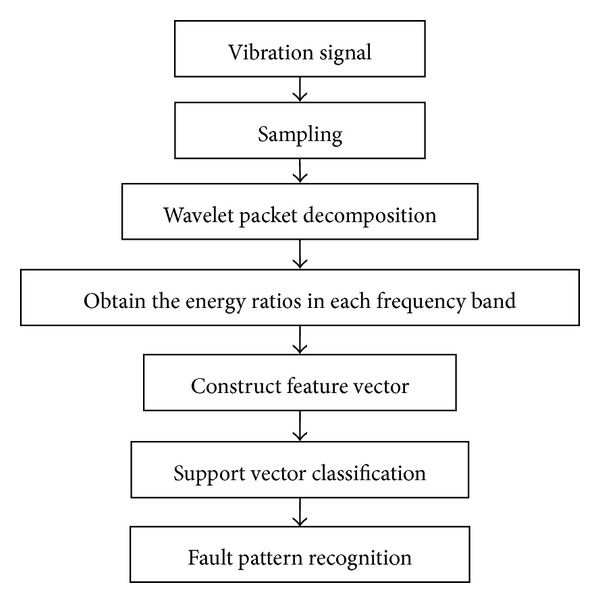
Fault pattern recognition flowchart.

The fault pattern recognition flowchart based on wavelet packet decomposition and SVC is shown in Figure 1. The wavelet packet is employed to decompose the vibration signal to obtain the energy ratio in each frequency band. Taking these energy ratios as feature vectors, we can detect faults from the determined fault type through the trained SVC. The detailed procedures are described in Sections 2.1 and 2.2.

### 2.1. Wavelet Packet Decomposition to Extract Feature Vector

#### 2.1.1. 3-Layer Decomposition for Each Signal Using Wavelet Packet

When decomposing the vibration signal using wavelet packet, the binary tree structure will be obtained. The final layer of the binary tree structure of the energy ratio in each frequency band can be obtained through calling the wenergy function. According to WPT theory, index (*i*, *j*) represents the *i*th layer and the *j*th node (*j* = 2^*i*^) as well as a certain signal component (frequency band). For example, (0, 1) represents the original signal, (1, 1) represents the low frequency wavelet packet decomposition coefficients of the first layer, and (1, 2) represents the high frequency wavelet packet decomposition coefficients of the same layer. For the 3-layer (*i* = 3) case, the corresponding node *j* = 1, 2,…, 8. Therefore, we have eight indexes (3, *j*) representing eight signal components (frequency bands).

#### 2.1.2. Obtaining the Energy Ratio in Each Frequency Band

Generally, the frequency bands are not arranged in accordance with the frequency order from low to high. When the original vibration signal is decomposed by wavelet packet (through the high-pass filter and downsampling procedure), the spectral sequence will be flipped. Therefore, to calculate the energy ratios, we should adjust the order of the corresponding frequency bands. For the 3-layer (*i* = 3) case, the exact frequency bands in accordance with the frequency from low to high should be (3, 1), (3, 2), (3, 4), (3, 3), (3, 7), (3, 8), (3, 6), and (3, 5) [23].

#### 2.1.3. Construct Feature Vector

The energy of the vibration component in each band will be changed when the faults of the mechanical system occurred. For the different faults, the energy ratios will be changed accordingly. Therefore, for the 3-layer (*i* = 3) case of wavelet packet decomposition, a feature vector **T** can be constructed by the eight energy ratios as

(1) $$\begin{array}{c} T=\left\{E_{31},E_{32},E_{33},E_{34},E_{35},E_{36},E_{37},E_{38}\right\}. \end{array}$$

Here, *E*_3*j*_ (*j* = 1, 2, 3,…, 8) represent all of the energy ratios for the 3rd layer and the *j*th node.

### 2.2. Fault Pattern Recognition Using SVC

The basic theory for SVC is summarized in this section [24].

Assume that a training set *S* is given by

(2) $$\begin{array}{c} S={\left\{x_{i},y_{i}\right\}}_{i=1}^{n}, \end{array}$$

where *x*_*i*_ ∈ *R* ^*n*^ and *y*_*i*_ ∈ {−1, +1}. The goal of SVMs is to find an optimal hyper plane such that

(3) $$\begin{array}{c} w^{T}x_{i}+b\geq 1,\;\text{for}\;\;y_{i}=+1,\\ w^{T}x_{i}+b\leq 1,\;\text{for}\;\;y_{i}=-1, \end{array}$$

where the weight vector *w* ∈ *R* ^*n*^ and the bias *b* is a scalar.

If the inequality in (3) holds for all training data, it will be a linearity separable case. Therefore, to find the optimal hyper plane, one can solve the following constrained optimization problem:

(4)  Minimize Φ(w)=12wTw Subject  to yi(wTxi+b)≥1, i=1,2,…,n.

If inequality in (3) does not hold for some data points in *S*, SVMs become linearly not separable. To find an optimal hyper plane, we have to solve the following constrained optimization problem:

(5)   Minimize  Φ(w)=12wTw+C∑i=1nξi  Subject  to   yi(wTxi+b)≥1−ξi,          ξi≥0, i=1,2,…,n.

By introducing a set of Lagrange multipliers *α*_*i*_, *β*_*i*_ for constraints, the problem becomes the one to find the saddle point of the Lagrangian. Therefore, the dual problem becomes

(6)  Minimize  Q(α)=∑i=1nαi−12∑i=1n∑j=1nαiαjyiyjxiTxj

(7)  Subject  to  ∑i=1nαiyi=0,

(8)          0≤αi≤C, i=1,2,…,n.

If 0 < *α*_*i*_ ≤ *C*, the corresponding data points are called support vectors (SVs).

SVMs map the input vector into a higher dimensional feature and, thus, can solve the nonlinear case. By choosing a nonlinear mapping function *φ*(*x*) ∈ *R* ^*m*^, where *M* > *N*, the SVM can construct an optimal hyper plane in the new feature space. *K*(*x*, *x*_*i*_) is the inner product kernel performing the nonlinear mapping into feature space as

(9) $$\begin{array}{c} K\left(x,x_{i}\right)=K\left(x_{i},x\right)=\varphi {\left(x\right)}^{T}\varphi \left(x_{i}\right). \end{array}$$

Therefore, the dual optimization problem becomes

(10) $$\begin{array}{c} \text{Minimize}\;Q\left(\alpha \right)=\sum_{i=1}^{n}\alpha_{i}-\frac{1}{2}\sum_{i=1}^{n}\sum_{j=1}^{n}\alpha_{i}\alpha_{j}y_{i}y_{j}K\left(x_{i},x_{j}\right) \end{array}$$

and the constraints are the same as shown in (7) and (8); the only requirement on the kernel *K*(*x*, *x*_*i*_) is to satisfy the Mercer's theorem [24]. Using Kernel functions, every data will be classified as

(11) $$\begin{array}{c} x\in \{\begin{array}{cc} \text{positive}\;\text{class}, & \text{if}\;g\left(x\right)>0\\ \text{negative}\;\text{class}, & \text{if}\;g\left(x\right)<0 \end{array} \end{array}$$

in which the decision function is

(12) $$\begin{array}{c} g\left(x_{i}\right)=y_{i}\left(\sum_{j=1}^{N}y_{j}\alpha_{j}K\left(x_{i},x_{j}\right)+b\right). \end{array}$$

The typical examples of kernel function are polynomial kernel, radial basis function (RBF) kernel, sigmoid kernel, and linear kernel. In many practical applications [25–27], the RBF kernel obtains the highest classification accuracy rate than other kernel functions. Therefore, the RBF kernel is employed in the present investigation.

Support vector machines were originally designed for binary classification. How to effectively extend it for multiclass classification is still an ongoing research issue. Currently there are several methods that have been proposed for multiclass classification, such as one-against-one, one-against-all, and directed acyclic graph (DAG). Hsu and Lin [28] gave a comparison of these methods and pointed out that the one-against-one method is more suitable for practical use than others. In the present, the one-against-one method is applied to detect the faults of bearings and gears.

## 3. Experimental Investigation

In this section, two typical fault diagnosis experiments for bearings and gears are given to testify the performance of the proposed hybrid approach.

### 3.1. Bearing Fault Diagnosis

**Figure 2:**
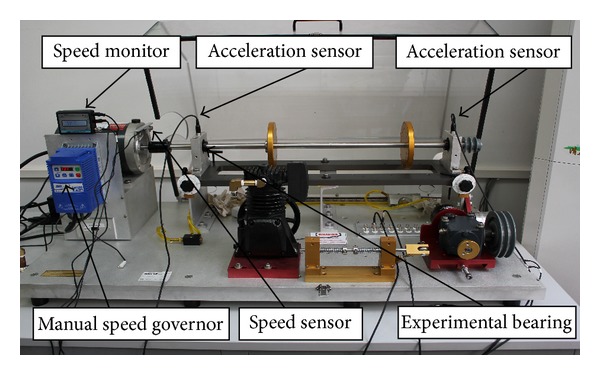
The MFS-MG experimental platform.

**Figure 3:**
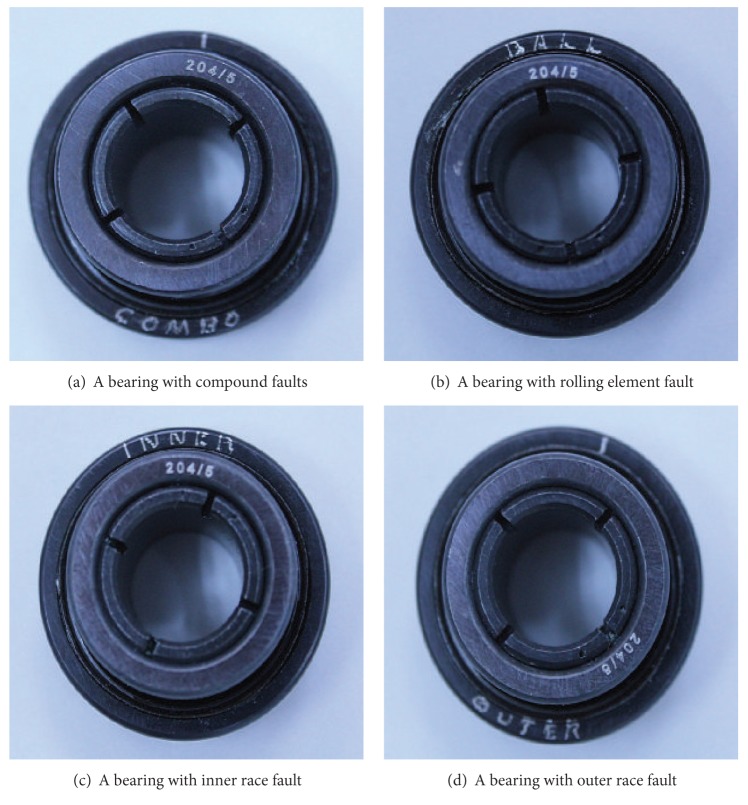
Bearings with typical faults.

The MFS-MG experimental platform for the bearing fault simulation [29] is shown in Figure 2. It includes speed monitor, manual speed governor, acceleration sensors, speed sensor, motor, spindle, bearings, and so forth. During the experiment, the data are acquired by an accelerometer mounted on the top of the bearing holder on left side. The vibration signals of the five fault types are collected, that is, normal case, rolling element fault, inner race fault, outer race fault, and compound fault (including inner race, outer race, and rolling element faults). The spindle speed is 1792 rpm and the end of the experimental bearing (see Figure 2) is free of loading. The bearings with typical faults are shown in Figure 3: the bearing model is ER-12K, the bearing pitch diameter is 33.4772 mm, the number of rolling element is 8, and the rolling element diameter is 7.9375 mm. Figures 3(a), 3(b), 3(c), and 3(d) show four fault cases, that is, a bearing with compound faults, a bearing with rolling element fault, a bearing with inner race fault, and a bearing with outer race fault, respectively.

**Figure 4:**
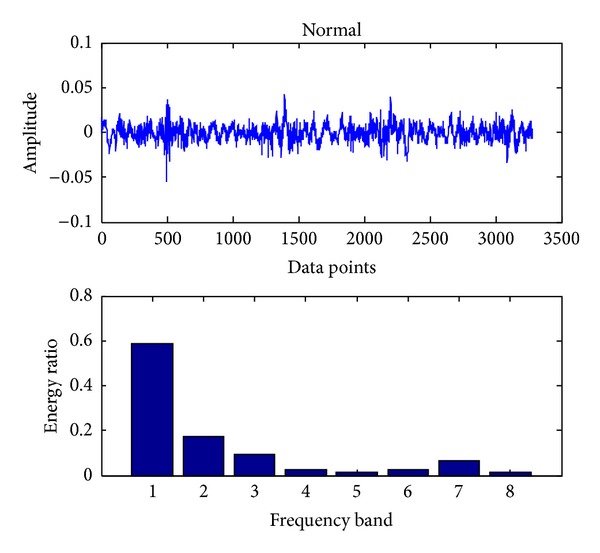
Normal case of a bearing.

**Figure 5:**
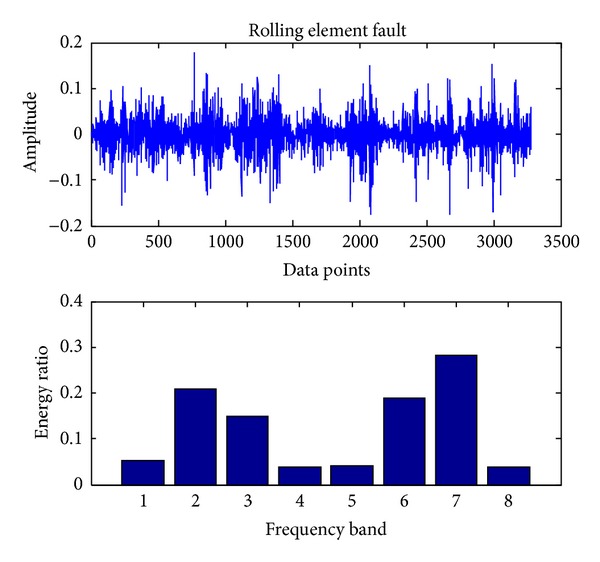
Rolling element fault.

**Figure 6:**
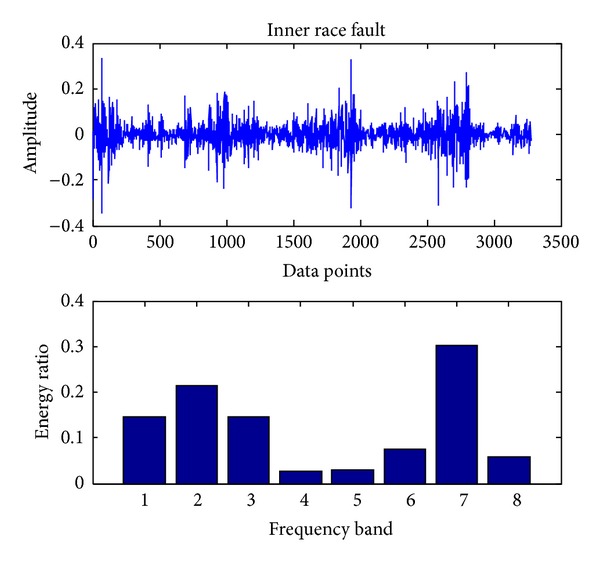
Inner race fault.

**Figure 7:**
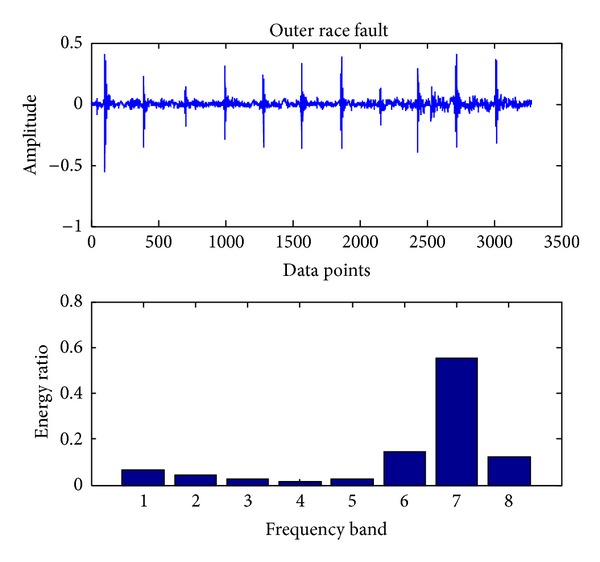
Outer race fault.

**Figure 8:**
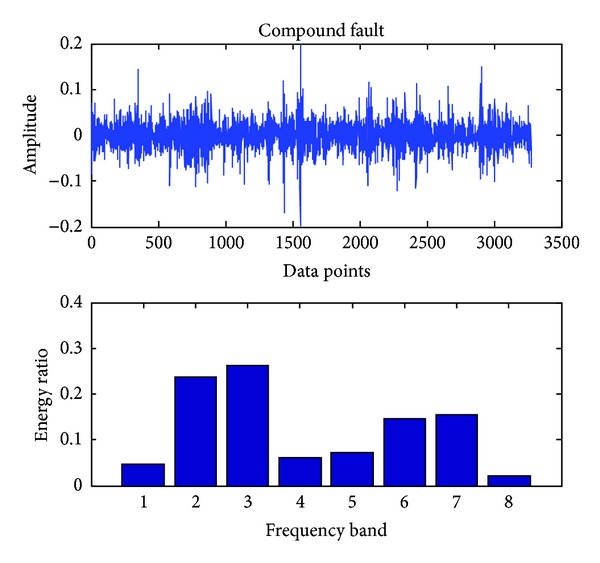
Compound fault of a bearing.

In the present, the sampling frequency is 25.6 k. 163800 data points (bearing vibration signal) are collected at one running in different cases and divided into 50 sections. Each signal contains 3276 data points. Db18 wavelet [23] is used to decompose each signal into three layers and gain 8 sub-bands in the final layer. The energy ratio of each band is obtained through calling the wenergy function in the wavelet toolbox of Matlab. Therefore, as shown in (1), a 1 × 8 vector (feature vector) is obtained. Figures 4, 5, 6, 7, and 8, respectively, represent the graph of the original signals and the corresponding eight energy ratios distribution maps for five cases including normal case and four fault cases.

For each of the five cases, we collect two bearing vibration signals (each signal contains 163800 data points) at two runnings. The first signal is employed to train the SVM and the second signal to be tested. According to the above description, 50 feature vectors can be extracted from each signal. Therefore, 50 feature vectors (samples) in the first running are served as training samples and the other 50 feature vectors (samples) in the second running are the fault samples to be tested (classified). Tables 1 and 2 give the first three training samples of the first signal and the first three test samples of the second signal. To represent the five cases numerically, we label the normal case, the rolling element fault, the inner race fault, the outer race fault, and the compound fault as 1 to 5, respectively. It points out that they are called standard labels.

In the present investigation, we adopt the SVM toolkit programmed by Franc and Hlavác of the Czech Technical University [30].

For general analysis, it is desirable to use normalized, nondimensional parameters. The normalized parameters also speed up the computational process. Therefore, prior to the training of the SVC model, all samples data are normalized to be bounded by [0, 1].

According to the SVC algorithm, the samples and label are

(13) {Xi,His}i=1l,Xi={E31,E32,E33,E34,E35,E36,E37,E38},for  His=y,

where **X**_*i*_ and *H*_*i*_^*s*^ are, respectively, the samples and label and *l* is the number of samples. From the above, the data for samples constitute a 50 × 8 matrix and the label lead to a 50 × 1 vector. Then, the first signal (50 segments) in all cases inputs the support vector machine for training. That means it includes two matrices, that is, the 250 × 8 matrix of samples and the 250 × 1 vector of label. After training, the second signal (50 segments) in all cases separately inputs the trained SVM for testing. Therefore, it includes a 50 × 8 vector of test samples. The predicted output (predicted label) will be obtained when the test is completed, and the recognition rate of every case can be obtained through the comparison of the predicted label with the standard labels. For each training and prediction, arg, *C* (arg = 1, *C* = 10, suggested by Franc and Hlavác [30]) and radial basis functions are selected as kernel argument, regularization constant, and kernel functions, respectively.

Table 3 shows the SVC results. From Table 3, we can see that the recognition rate of rolling element fault is 86% and the inner race fault is 96%. For the other three cases, that is, the normal case, the outer race fault, and compound fault, we obtain high performance results with 100% accuracy.

### 3.2. Gear Fault Diagnosis

**Figure 9:**
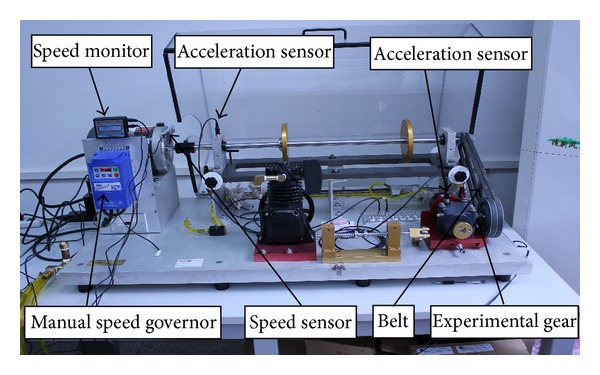
The MFS-MG experimental platform.

**Figure 10:**
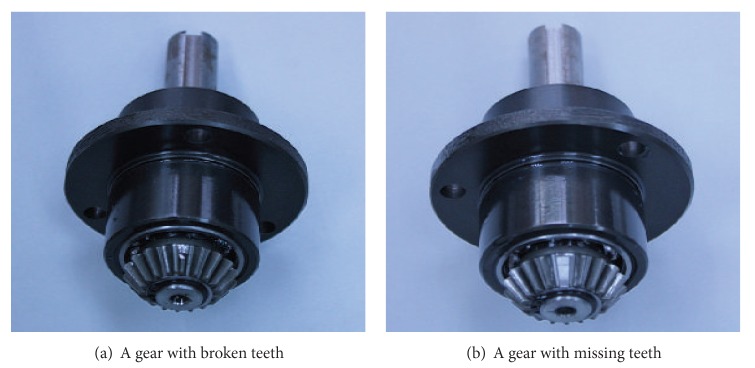
Typical fault gear.

The MFS-MG experimental platform for the gear fault simulation [29] is shown in Figure 9. Compared to the bearing fault simulator, the gear fault simulator has a slight difference; that is, a gearbox and a transmission belt are added. In addition, two normal bearings are installed at both the bearing holder on left side and the bearing holder on right side. During the experiment, the data are acquired by an accelerometer mounted on the top of the gearbox. The vibration signals of four cases are collected, that is, normal case, broken teeth, wears, and gear missing teeth. The spindle speed is 1764 rpm and the end of the gearbox is free of loading, the gearbox transmission ratio is 1.5 : 1, the gear teeth is 18, pitch diameter is 28.575 mm, and helix angle is 33°41′. Two fault cases, that is, a gear with broken teeth and a gear with missing teeth, are shown in Figures 10(a) and 10(b), respectively.

**Figure 11:**
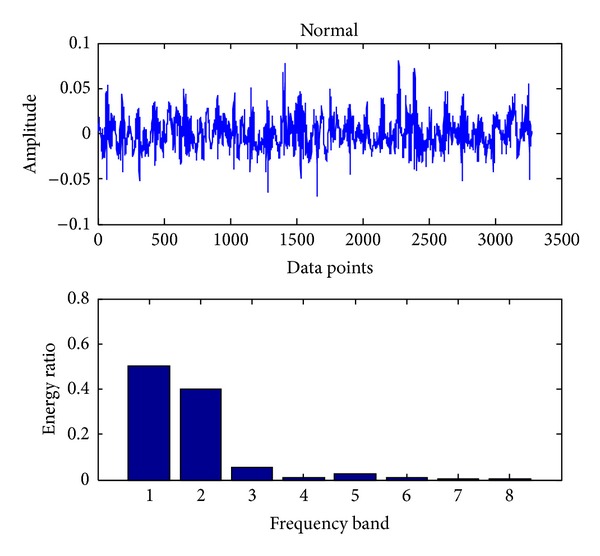
Normal case of a gear.

**Figure 12:**
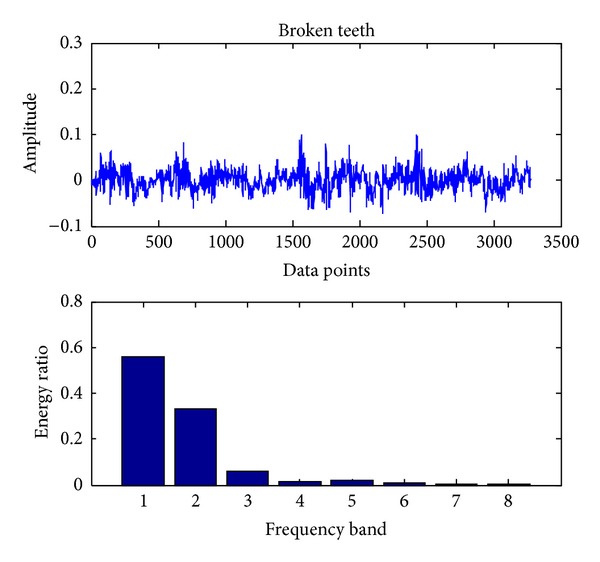
Gear broken teeth.

**Figure 13:**
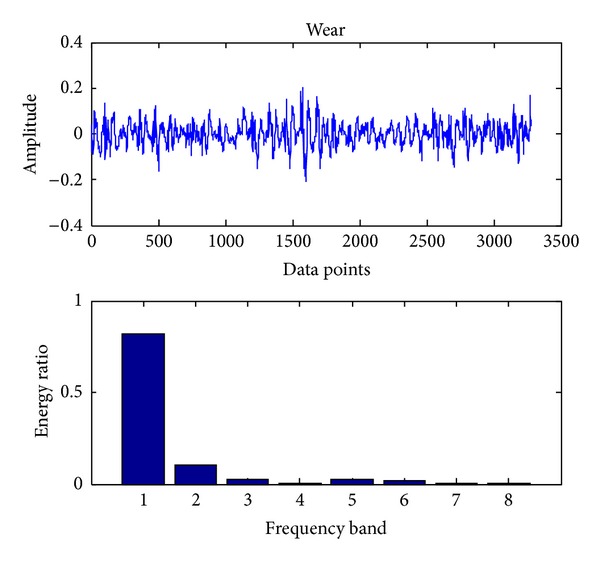
Gear wear.

**Figure 14:**
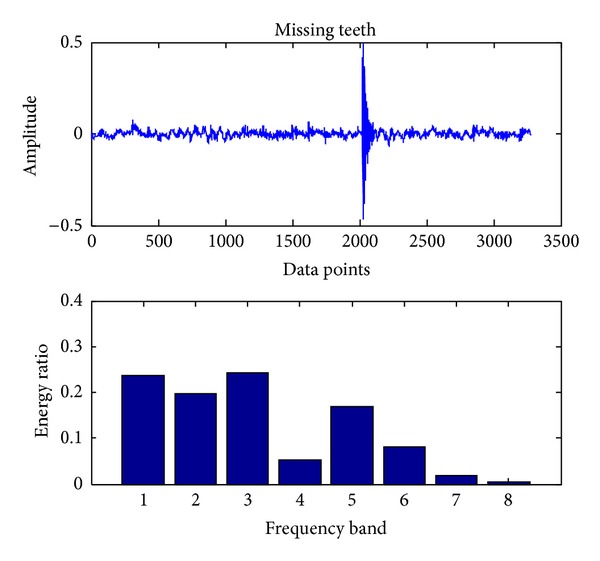
Gear missing teeth.

Similar to bearing fault diagnosis, we use the same sampling frequency and also 163800 data points (gear vibration signal). To detect the faults using SVC, we proceed with the same procedures as shown in Section 3.1. The original signals and the corresponding eight energy ratios distribution maps for four cases, including normal case and three fault cases (broken teeth, wear and missing teeth), are shown in Figures 11, 12, 13, and 14, respectively.

The first three training samples of the first signal and the first three test samples of the second signal are shown in Tables 4 and 5, respectively. To represent the four cases numerically, we define the standard labels, that is, the normal case, the broken teeth case, the wear case, and the missing teeth case as 1 to 4, respectively.

The SVC results are shown in Table 6. The recognition rates for the normal case, the broken teeth, the wear, and the missing teeth are 94%, 96%, 100%, and 100%, respectively.

Based on the above experimental investigations, the proposed hybrid approach is reasonably effective for detecting different kinds of faults in both bearings and gears.

## 4. Conclusion

This paper proposes a hybrid approach using wavelet packet and SVC to classify faults for bears and gears. The collected vibration signals are directly employed as inputs without any pretreatment. The signals are decomposed by wavelet packet and the energy ratios of all frequency bands are calculated to construct the feature vectors so as to train and test the support vector machines to predict the fault type of bearings and gears. Experimental investigations for bearing fault diagnosis and gear fault diagnosis are made using MFS-MG experimental platform (the bearing fault simulator and the gear fault simulator). The results show that the proposed hybrid approach is effective to the rotating and the transmission structures. Moreover, the present approach has a good recognition rate not only for a single fault but also for the compound fault.

## Figures and Tables

**Table 1:** Bearing training samples.

Cases	Energy ratios in each frequency band							
	1	2	3	4	5	6	7	8
Normal	0.5862	0.1738	0.0930	0.0252	0.0165	0.0263	0.0638	0.0155
	0.4725	0.2200	0.1097	0.0295	0.0253	0.0338	0.0899	0.0193
	0.5887	0.1845	0.0815	0.0210	0.0148	0.0219	0.0707	0.0168

Rolling element fault	0.0519	0.2047	0.1484	0.0389	0.0457	0.1648	0.3067	0.0390
	0.0556	0.1537	0.1239	0.0471	0.0544	0.1664	0.3229	0.0761
	0.0516	0.1457	0.0830	0.0415	0.0533	0.1692	0.4006	0.0551

Inner race fault	0.1458	0.2153	0.1452	0.0272	0.0292	0.0757	0.3030	0.0586
	0.1691	0.2262	0.1234	0.0375	0.0446	0.0780	0.2779	0.0433
	0.0857	0.1596	0.1210	0.0238	0.0343	0.1040	0.3967	0.0749

Outer race fault	0.0664	0.0448	0.0259	0.0129	0.0277	0.1449	0.5550	0.1224
	0.0636	0.0410	0.0165	0.0111	0.0215	0.1190	0.5888	0.1386
	0.0740	0.0311	0.0141	0.0090	0.0249	0.1745	0.5404	0.1321

Compound fault	0.0457	0.2360	0.2616	0.0623	0.0731	0.1457	0.1544	0.0213
	0.0464	0.1528	0.1851	0.0691	0.0695	0.1925	0.2556	0.0291
	0.0553	0.1896	0.2769	0.0748	0.0770	0.1263	0.1774	0.0228

**Table 2:** Bearing test samples.

Cases	Energy ratios in each frequency band							
	1	2	3	4	5	6	7	8
Normal	0.5318	0.2206	0.0832	0.0256	0.0198	0.0292	0.0718	0.0180
	0.5826	0.1843	0.0945	0.0200	0.0178	0.0232	0.0628	0.0148
	0.5623	0.1877	0.0938	0.0209	0.0221	0.0251	0.0722	0.0160

Rolling element fault	0.0553	0.1541	0.1091	0.0403	0.0624	0.1990	0.3271	0.0528
	0.1066	0.2799	0.0772	0.0219	0.0339	0.1315	0.3022	0.0469
	0.0364	0.0993	0.0677	0.0355	0.0365	0.1707	0.4851	0.0686

Inner race fault	0.1295	0.1626	0.1031	0.0294	0.0504	0.0944	0.3612	0.0693
	0.1548	0.2436	0.1546	0.0434	0.0392	0.0749	0.2385	0.0511
	0.1403	0.1962	0.1596	0.0286	0.0309	0.0794	0.3197	0.0452

Outer race fault	0.0428	0.0640	0.0319	0.0170	0.0190	0.1508	0.5503	0.1243
	0.0304	0.0416	0.0238	0.0117	0.0230	0.1421	0.5469	0.1805
	0.0598	0.0867	0.0330	0.0135	0.0196	0.1422	0.5166	0.1285

Compound fault	0.0388	0.1612	0.1681	0.0670	0.0679	0.2692	0.1964	0.0313
	0.0533	0.1979	0.2272	0.0811	0.0737	0.1538	0.1905	0.0224
	0.0636	0.2239	0.1841	0.0691	0.0629	0.1777	0.1941	0.0246

**Table 3:** Bearing recognition results.

Cases	Training samples	Testing samples	Classification label	Recognition rate
Normal	50	50	1	100%
Rolling element fault	50	50	2	86%
Inner race fault	50	50	3	96%
Outer race fault	50	50	4	100%
Compound fault	50	50	5	100%

**Table 4:** Gear training samples.

Cases	Energy ratios in each frequency band							
	1	2	3	4	5	6	7	8
Normal	0.5022	0.3977	0.0530	0.0077	0.0250	0.0098	0.0034	0.0012
	0.4511	0.4109	0.0716	0.0079	0.0407	0.0128	0.0038	0.0013
	0.4911	0.3799	0.0651	0.0080	0.0360	0.0150	0.0037	0.0011

Broken teeth	0.5591	0.3319	0.0615	0.0152	0.0224	0.0065	0.0029	0.0007
	0.6000	0.2979	0.0578	0.0130	0.0226	0.0060	0.0023	0.0005
	0.5589	0.3253	0.0686	0.0146	0.0216	0.0073	0.0031	0.0006

Wear	0.8194	0.1025	0.0226	0.0059	0.0266	0.0206	0.0018	0.0006
	0.8289	0.0902	0.0178	0.0056	0.0380	0.0173	0.0016	0.0005
	0.8080	0.1084	0.0209	0.0061	0.0391	0.0155	0.0018	0.0003

Missing teeth	0.2358	0.1971	0.2427	0.0528	0.1688	0.0814	0.0177	0.0035
	0.1239	0.2404	0.1706	0.0746	0.2052	0.1422	0.0387	0.0044
	0.2064	0.3353	0.1174	0.0537	0.1634	0.1051	0.0141	0.0046

**Table 5:** Gear test samples.

Cases	Energy ratios in each frequency band							
	1	2	3	4	5	6	7	8
Normal	0.5055	0.3774	0.0664	0.0074	0.0285	0.0099	0.0034	0.0015
	0.4699	0.3989	0.0656	0.0089	0.0345	0.0160	0.0048	0.0014
	0.4881	0.4068	0.0555	0.0088	0.0257	0.0099	0.0038	0.0014

Broken teeth	0.5709	0.3092	0.0712	0.0152	0.0235	0.0068	0.0027	0.0006
	0.5425	0.3387	0.0668	0.0163	0.0238	0.0069	0.0038	0.0012
	0.5727	0.3254	0.0645	0.0111	0.0176	0.0059	0.0022	0.0006

Wear	0.8120	0.1040	0.0188	0.0055	0.0330	0.0255	0.0009	0.0003
	0.8267	0.0975	0.0175	0.0055	0.0301	0.0215	0.0009	0.0003
	0.8521	0.0831	0.0190	0.0061	0.0236	0.0141	0.0008	0.0010

Missing teeth	0.1902	0.3251	0.1063	0.0116	0.1712	0.1581	0.0334	0.0041
	0.1932	0.3082	0.0844	0.0244	0.1607	0.1909	0.0299	0.0083
	0.2200	0.2558	0.1340	0.0092	0.1816	0.1727	0.0212	0.0055

**Table 6:** Gear recognition results.

Cases	Training samples	Testing samples	Classification label	Recognition rate
Normal	50	50	1	94%
Broken teeth	50	50	2	96%
Wear	50	50	3	100%
Missing teeth	50	50	4	100%
